# Glances at the Hospitals

**Published:** 1902-11-08

**Authors:** 


### GLANCES AT THE HOSPITALS.

### NATIONAL HOSPITAL FOR CONSUMPTION, IRELAND.

THIS sanatorium is situated near Newcastle, in the county of Wicklow. It contains 66 beds, and was opened in 1896 for the treatment of poor patients suffering from pulmonary consumption. The treatment adopted is that of the open-air system, and the total number of patients so treated has been 465, including 39 cases in the hospital on the date of the report. As might be expected, the hospital has only a very small endowment, and donations and subscriptions, on which the institution is almost entirely dependent, would be gratefully received by the Honorary Secretary at 5 Leinster Street, Dublin. The managers state that the medical report is distinctly encouraging, and that a better average of marked improvement in the patients has been obtained this year than in the previous year. They add that, "while time alone can demonstrate the permanence of the cure in any particular instance, the history of the cases treated justifies the confidence in the curability of consumption which is now so generally expressed with the highest medical sanction." There can be no doubt about the truth of this statement; and the results obtained in our sanatoria would be still more satisfactory if sufficient beds were in existence, and sufficient funds to keep them up, to enable applicants to be treated in the earliest stage of the disease. As it is the unfortunate man who cannot meet the payments asked for at a private sanatorium has to wait his turn for admission to a public one, and this too often means many months' delay, during which time impure air, insufficient food, and perhaps an insanitary occupation, have advanced the disease to such a point that a permanent cure is almost hopeless. If these points could be driven into the minds of the rich and charitably-inclined people of Ireland, surely sufficient subscriptions and donations would be forthcoming to enable the board to open additional blocks and to keep them going. Much has already been done in this direction. Mr. Wellington Darley gave the hospital £1,000, which has been expended in the erection of a dining hall to accommodate 100 patients. A bequest of £6,000 enabled the board to erect a block containing 6 wards, which will add 26 beds to the sanatorium. The board wisely decided to erect this block of wood, as being cheaper and more quickly put up. The total number of patients treated last year was 164, and the average residence of the patients was 13⅘ weeks. Of the total number under treatment, we learn that 10 were so much improved that a permanent arrest of the disease was probable; 32 were much improved, and a fair number of these also afforded hope of permanent arrest; 53 were improved, the progress of the disease being more or less checked; 14 remained in about the same state as on admission; 5 became worse, and 2 died. The remaining 9 cases had the physical signs of consumption, but the tubercle bacillus was not identified. Of these, 3 left the hospital with no signs of disease, 2 were much improved, 3 were improved, and 1 unchanged.

### ROCHDALE INFIRMARY AND DISPENSARY.

THE ordinary income amounted to £2,898, and the ordinary expenditure to £3,204. The deficit for the year added to that carried forward from the previous year amounts to £674, and the "Board feel that the institution does not receive the financial support which it is entitled to from the working population of the district." We notice that the mill and workshop contributions amount to £794. At first sight this seems a fair sum; but when we think that the population of Rochdale exceeds 70,000 and abounds with factories, it is certain that the above-named sum ought to be greatly increased by that class, for whom the infirmary chiefly exists. We trust that the next annual report will show that the appeal of the managers has not been made in vain. Failing a reasonable response, it is certain that the usefulness of the institution will be curtailed. Even now we are told that improvements in the laundry department are required, but they cannot be proceeded with for want of funds. The number of in-patients admitted was 427, the daily average number of beds occupied was 33, the average stay of each patient in the hospital was 30 days. It is stated that this period is a decrease upon that common in recent years, and we believe it might be still further reduced, and so decrease expenses or enable a larger number of in-patients to be treated. The death rate was 6·5 per cent. We failed to find any statement showing the cost per head or per bed occupied. Of the total number under treatment, 398 were discharged cured, 44 were discharged relieved, 8 were discharged for various reasons, 31 died, and 40 remained under treatment. The medical and surgical tables are properly arranged.

### EVELINA HOSPITAL FOR SICK CHILDREN, SOUTHWARK BRIDGE ROAD, S.E.

THE ordinary income amounted to £5,154, and the ordinary expenditure to £6,548. At the beginning of the year there was a balance of £433 in the bankers' hands, and a sum of £1,500 was transferred from the deposit account to the current account. The financial statements, so far as they refer to the administration of the hospital, are meagre, or rather non-existent. We are not told the cost per head, nor the cost per patient, nor the cost per bed occupied. Such omissions ought hardly to occur, and we hope they will be corrected in next report. On January 1st the hospital contained 36 patients, and 981 were admitted during the year, making a total of 1,017. Of these 775 were discharged either cured or relieved, 12 were discharged on other grounds, 173 died, and 57 remained in the hospital at the end of the year. The average time each child remained in the hospital was 22 days, and the average daily number of beds occupied was 61. At the out-patients' department 18,190 new patients and casualties were treated, and these gave a daily average of 115 attendances. These statistics prove that an enormous amount of good work is being done by the hospital to its district and in a smaller degree to London and the country. Of the new cases admitted 737 were local, 122 from other parts of London, and 52 from the country. The medical and surgical tables give the numbers treated for the various diseases and the results of treatment. Of 127 cases of pneumonia 101 were cured, 24 died, and 2 were discharged for other reasons. Of 12 cases of enteric fever 11 were cured and 1 died. A table is inserted showing the cause of death and length of time in the hospital of every one of the 173 deaths. Anæsthetics were administered 1,250 times, but the agent used is, unfortunately, not stated.

### EVERSFIELD HOSPITAL, ST. LEONARDS.

THE income amounted to £1,864, and the expenditure to £2,129, showing a deficit of £265 on the year's working. The total liabilities were over £1,000, but the managers point out that this large sum is mainly the result of the alterations and repairs to the building, and they add their belief that their "pressing needs will elicit a generous response, and that the close of this year will find us free from debt." There were 34 patients in the hospital at the beginning of the year, and 196 were admitted during it, making a total of 230. The average number resident was 31, and the death rate was 6·10 per cent. The hospital is for the treatment of diseases of the respiratory organs, and for this purpose the climate of St. Leonards-on-Sea is very suitable. The number prescribed for in the out-patients' department was 1,495, and the amount contributed by these patients was £47. No charge is made; but the managers think that gratuitous medical advice has a pauperising effect, and they expect that everyone who is able will contribute something. Patients are received from all parts of the kingdom, irrespective of creed. In the general wards the payment is 13s. a week, with a subscribers' ticket, or 17s. without a ticket. There are eight private wards, the charges varying from £2 2s. to £5 5s. a week. There are no medical tables, and no statement of results is given.

